# A chitinase-like protein from *Sarcoptes scabiei* as a candidate anti-mite vaccine that contributes to immune protection in rabbits

**DOI:** 10.1186/s13071-018-3184-y

**Published:** 2018-11-20

**Authors:** Nengxing Shen, Haojie Zhang, Yongjun Ren, Ran He, Jing Xu, Chunyan Li, Weimin Lai, Xiaobin Gu, Yue Xie, Xuerong Peng, Guangyou Yang

**Affiliations:** 1 Department of Parasitology, College of Veterinary Medicine, Sichuan Agricultural University, Chengdu, 611130 China; 2 Animal Breeding and Genetics Key Laboratory of Sichuan Province, Chengdu, 610066 China; 3 Department of Chemistry, College of Life and Basic Science, Sichuan Agricultural University, Chengdu, 611130 China

**Keywords:** *Sarcoptes scabiei*, Scabies, Chitinase-like protein 5, Recombinant protein, Vaccine

## Abstract

**Background:**

Scabies is caused by *Sarcoptes scabiei* burrowing into the stratum corneum of the host’s skin and is detrimental to the health of humans and animals. Vaccines are an attractive alternative to replace the acaricides currently used in their control.

**Methods:**

In the present study, the *S. scabiei* chitinase-like protein 5 (SsCLP5) was characterized and recombinant SsCLP5 (rSsCLP5) was evaluated as a candidate vaccine protein for anti-mite protection in rabbits. The expression, characterization and immunolocalization of SsCLP5 were examined. Vaccination experiments were performed on three test groups (*n* = 12 per group) immunized with purified rSsCLP5. Control groups (*n* = 12 per group) were immunized with PBS, QuilA saponin or empty vector protein. After challenge, the inflammatory reaction and skin lesions were graded and rSsCLP5 indirect ELISA was used to detect antibody IgG levels in serum samples at the time of vaccination and post-challenge.

**Results:**

The results showed that rSsCLP5 had high immunoreactivity and immunogenicity. In *S. scabiei*, SsCLP5 had a wide distribution in the chewing mouthpart, legs and exoskeleton, especially the outer layer of the exoskeleton. Vaccination with rSsCLP5 resulted in 74.3% (26/35) of rabbits showing no detectable lesions after challenge with *S. scabiei*.

**Conclusions:**

Our data demonstrate that rSsCLP5 is a promising candidate for a recombinant protein-based vaccine against *S. scabiei*. This study also provides a method for studying scabies vaccine using rabbit as an animal model and a basis for screening more effective candidate proteins.

**Electronic supplementary material:**

The online version of this article (10.1186/s13071-018-3184-y) contains supplementary material, which is available to authorized users.

## Background

Scabies is a highly contagious parasitic disease caused by the etiological agent *Sarcoptes scabiei*, which burrows into the stratum corneum of the host. Scabies or sarcoptic mange presents an enormous threat to the health of humans and animals worldwide [1, 2], occurring in more than 100 species of mammals [3] and causing clinical symptoms such as skin inflammation, itching and skin lesions [4]. Scabies occurs extensively in indigenous populations [5] and in the poorest areas of developing countries [6]. The uncontrolled spread of scabies or sarcoptic mange results in severe mortality, which has significant impacts in terms of welfare and economic loss [7–9]. Furthermore, in tropical climates, infection with *Streptococcus pyogenes* or *Staphylococcus aureus* often occurs, resulting in serious pyoderma of the skin lesions [10, 11].

At present, acaricides are used as a control measure for combating mite infestation; however, they can be toxic to humans and animals. For instance, neurotoxicity has been reported in children with widespread skin damage following treatment with benzyl benzoate [12] or lindane [13, 14]. Additionally, side effects have also been reported in dogs treated with moxidectin [15]. Furthermore, the risk of development of drug resistance in scabies mite due to intensive use of acaricides cannot be entirely ruled out and requires thorough consideration [16–18]. Moreover, acaricide residues have harmful effects on health and threaten the environment. An effective anti-mite vaccine, in contrast, has the potential to protect people and animals more efficiently in terms of safety, environmental friendliness and economic costs.

*Sarcoptes scabiei* infestation can induce both innate and adaptive immune responses in the host [19, 20]. Protective immune responses to mite infestation have been described [21–23], with findings suggesting that it is possible to develop a vaccine to control the scabies mite. To date, some vaccination studies have been published [22, 24]; however, no promising anti-mite vaccine candidate protein has been identified. The completed analysis of the genome [25], transcriptome [26] and proteome [27] of *S. scabiei* provides a basis for screening more effective candidate vaccine proteins.

Chitinase-like proteins (CLPs) and chitinases are a family of mediators increasingly associated with infection, T cell-mediated inflammation, wound healing, allergy and asthma [28]. CLPs are homologous to chitinases, both of which belong to the glycoside hydrolase family 18, but lack the ability to degrade chitin. Both play an important role in T-helper type 2 (Th2)-driven responses and possibly contribute to the repair process during inflammation [29–31]. In some parasitic infections [32–34], increased levels of chitinases and CLPs may contribute to the host’s defense in mammals.

In this study, we describe the identification, characterization and immunolocalization of SsCLP5, a candidate protein for an anti-mite vaccine, and evaluate the potential of the rSsCLP5 protein in a vaccination trial for mite infestation in rabbits.

## Methods

### Animals and sources

Twenty New Zealand rabbits were purchased from Chengdu Tatsuo Biological Technology Co. Ltd. (Chengdu, China) and infested with *Sarcoptes scabiei* for a month. *Sarcoptes scabiei* was harvested by the Department of Parasitology, College of Veterinary Medicine, Sichuan Agricultural University. In brief, live mites including larvae, nymphs and adults were collected and isolated from severely affected rabbits by exposing the infested crust to 37 °C for 2 h. Partial mites were used for RNA and protein extraction and the remaining mites were used for a challenge test in a vaccination trial. RNA isolation from mites was performed using RNAprep pure Tissue Kit (TIANGEN, Beijing, China) and RNA was transcribed into cDNA using RevertAid First Strand cDNA Synthesis Kit (Thermo Fisher Scientific, Vilnius, Lithuania). Total crude protein was extracted from mites using Total Protein Extraction Kit (BestBio, Shanghai, China). cDNA and total crude protein were stored at -70 °C until assay.

### Expression and purification of rSsCLP5

Based on genomic data (GenBank: JXLN01012673.1) and proteomics data (GenBank: KPM08736.1), we identified the *S. scabiei* chitinase-like protein 5 (SsCLP5) and amplified regions showing high sequence homology with other species according to the results of epitope analysis. Primers for amplification were designed using Primer 5.0 software and were as follows: forward (5'-CGG GAT CCA TGC AAG AGC TTC GTA A-3'), with a *Bam*HI restriction site (underlined), and reverse (5'-CCC TCG AGA TCA TAG AAG ATC ATA GAA AT-3'), with an *Xho*I restriction site (underlined; Invitrogen, Beijing, China). SsCLP5 was amplified using the following PCR cycling conditions: 94 °C for 5 min; 30 cycles of amplification at 94 °C for 45 s, 55 °C for 45 s, and 72 °C for 45 s; followed by a final extension at 72 °C for 10 min. The fragment was cloned into the pET-32a (+) expression vector (Invitrogen), which was transformed into *Escherichia coli* BL21 (DE3) cells. Protein expression was induced with 1 mM isopropyl-β-D-thiogalactoside (IPTG) at 37 °C for 6 h. Recombinant *S. scabiei* chitinase-like protein 5 (rSsCLP5) was purified using a Ni-NTA His-tag affinity chromatography kit (Bio-Rad Laboratories, California, USA), according to the manufacturer’s instructions.

### Sequence analysis

DNAMAN software was employed to compare sequence similarity between homologous genes. SsCLP5 was analysed using the online software SignalP 4.1 (http://www.cbs.dtu.dk/services/SignalP/), Transmembrane Prediction Server (http://www.sbc.su.se/~miklos/DAS/) and TargetP (http://www.cbs.dtu.dk/services/TargetP/) to assess potential signal peptides, transmembrane regions and subcellular localization, respectively. ExPasy (http://web.expasy.org/protparam/) was used to calculate predicted molecular weight and pI values. Homologous proteins were found in the NCBI database and comparative analysis was performed using the online software Clustal W2 (http://www.ebi.ac.uk/tools/msa/clustalw2/). Finally, we used MEGA 5.05 software for adjacent structure analysis of system evolution, and to build the evolutionary tree [35, 36].

### Western blotting

Samples (40 μl of protein and 10 μl loading buffer) were boiled for 10 min. Protein samples were separated by 12% SDS-PAGE and transferred onto a nitrocellulose membrane for 35 min in an electrophoretic transfer cell (Bio-Rad Laboratories). Membranes were washed three times for 5 min in TBST (20 mM Tris-HCl, 150 mM NaCl, 0.05% [v/v] Tween 20, pH 7.4), blocked with 5% skim milk for 2 h, and then incubated overnight at room temperature with rabbit serum (diluted 1:100 with 0.01 M PBS). Next, the membranes were washed four times for 5 min each in TBST, then incubated with horseradish peroxidase (HRP)-conjugated goat anti-rabbit antibody (diluted 1:1000) for 2 h. The membranes were washed four times with PBS, and protein signals detected using diaminobenzidine reagent (TIANGEN, Beijing, China).

### Immunolocalization

Adult mites were fixed in 1% molten agarose shortly after collection. The solid agarose containing the mites was then embedded in paraffin wax and cut into sections (5 μm) with a microtome. The sections were baked in a 60 °C oven for 2 h, dewaxed in xylene twice for 7 min each, in 100% ethanol twice for 3 min each, in 95% ethanol for 3 min, in 85% ethanol for 3 min and in 75% ethanol for 3 min, and were then rinsed with distilled water for 8 min. To inactivate endogenous peroxidase, the sections were incubated in blocking buffer (3% H_2_O_2_ in PBS) for 20 min at 37 °C. Next, heat-induced epitope retrieval was accomplished by immersing sections in 0.01 M sodium citrate buffer (pH 6.0) at 95 °C for 20 min. The sections were incubated in blocking buffer (5% bovine serum albumin in PBS) for 1 h at room temperature before incubation overnight at 4 °C with specific rabbit anti-rSsCLP5 antibodies (preparation of rSsCLP5 polyclonal antibody as described previously [37]) covering the sections (diluted 1:100 in PBS). After washing three times with PBS, the sections were incubated with fluorescein isothiocyanate (FITC) goat anti-rabbit IgG (H+L; diluted 1:100; EarthOx, LLC, San Francisco, CA, USA) in 0.1% Evans Blue for 1 h at 37 °C in the dark. Finally, sections were viewed with a microscope. In this experiment, the negative controls consisted of pre-immune rabbit serum antibody instead of specific antibodies.

### Vaccination trial

A total of 72 three-month-old New Zealand rabbits (36 females and 36 males) were prepared for the vaccination trial by Chengdu Tatsuo Biological Technology Co. Ltd. Rabbits weighed 2.3 ± 0.2 kg at the beginning of the experiment and were immunized twice at a 14-day interval by subcutaneous injection of the neck. Rabbits were randomly divided into six groups of 12 rabbits each. Group one was inoculated with 1 ml 0.01 M PBS (137 mM NaCl, 2.7 mM KCl, 10 mM Na_2_HPO_4_, 2 mM KH_2_PO_4_, pH 7.4) as unvaccinated controls; group two was inoculated with 1 ml Quil-A saponin adjuvant (Sigma-Aldrich, St. Louis, MO, USA) at a concentration of 1 mg/ml (dissolved in PBS) as adjuvant controls; group three was inoculated with 100 μg (initial injection) and 200 μg (second injection) purified protein from the pET32a (+) empty vector with 1 ml Quil-A saponin at a concentration of 1 mg/ml (dissolved in PBS) as vector protein controls; and group four was immunized with 100 and 200 μg purified rSsCLP5 protein with 1 ml Quil-A saponin at a concentration of 1 mg/ml (dissolved in PBS) for the first and second immunizations, respectively. Group five and group six received the same immunization as group four and served as biological replicates. Two weeks after the second vaccination, each rabbit was challenged with approximately 2000 live mites on the two hind feet. In order to ensure proper and adequate infectivity, the live mites (larvae, nymphs and adults) were subjected to challenge test immediately after collection and counting under the microscope. The foot challenge area was chosen because mange lesions in naturally infested rabbits are most frequently initially observed in the hind limbs. Serum samples were obtained prior to vaccination and every week during vaccination and challenge until week 4 post-challenge. All sera samples were stored at -20 °C until use. Skin lesions of the hind legs were photographed weekly after the challenge. Mange lesion areas and the body weights were measured during the vaccination trial on a weekly basis.

### Lesion score and mite burden

After the challenge, skin lesions caused by the mites were assessed at weekly intervals from weeks 1 to 4 post-infestation. The lesion areas were photographed and measured using a caliper. The inflammatory reaction and skin lesions were graded as follows: 0, no inflammatory reaction; 1, mild inflammatory reaction; 2, severe inflammatory reaction; 3, lesions first observed on the limbs (lesions ≤ 7.75 cm^2^); 4, lesions of 7.75–15.5 cm^2^ (including 15.5 cm^2^) and 5, lesions of 15.5–31 cm^2^ [22, 24]. Four weeks after the challenge, all tested rabbits were euthanized and the crusts from the hind limbs were collected and digested with 10% KOH. Then, the dead mites were concentrated by floating with saturated sucrose solution and counted under the light microscope [38].

### Antibody responses

A checkerboard titration study was carried out to determine the optimal conditions for the rSsCLP5 and serum [39]. ELISA procedures were performed as previously described [37]. We used the rSsCLP5 indirect ELISA to detect antibody IgG levels in serum samples at the time of vaccination and post-challenge.

### Data analysis

All analyses were performed with R Statistical Environment [40], with confidence intervals at 95% (α = 0.050). All graphs were generated in GraphPad Prism version 5.0 (GraphPad Software). Statistical differences between groups were determined using IBM SPASS statistics 19.0 (SPASS Software). Analyses of variance for repeated measures for each dependent variable (IgG levels, lesion grades, lesion scores and weight) were performed using the *ez* package [41]. Data were analyzed using immunization group and time as fixed variables and the rabbit as a random variable to account for repeated measure variability.

## Results

### Identification and sequence analysis of SsCLP5

**Fig. 1:**
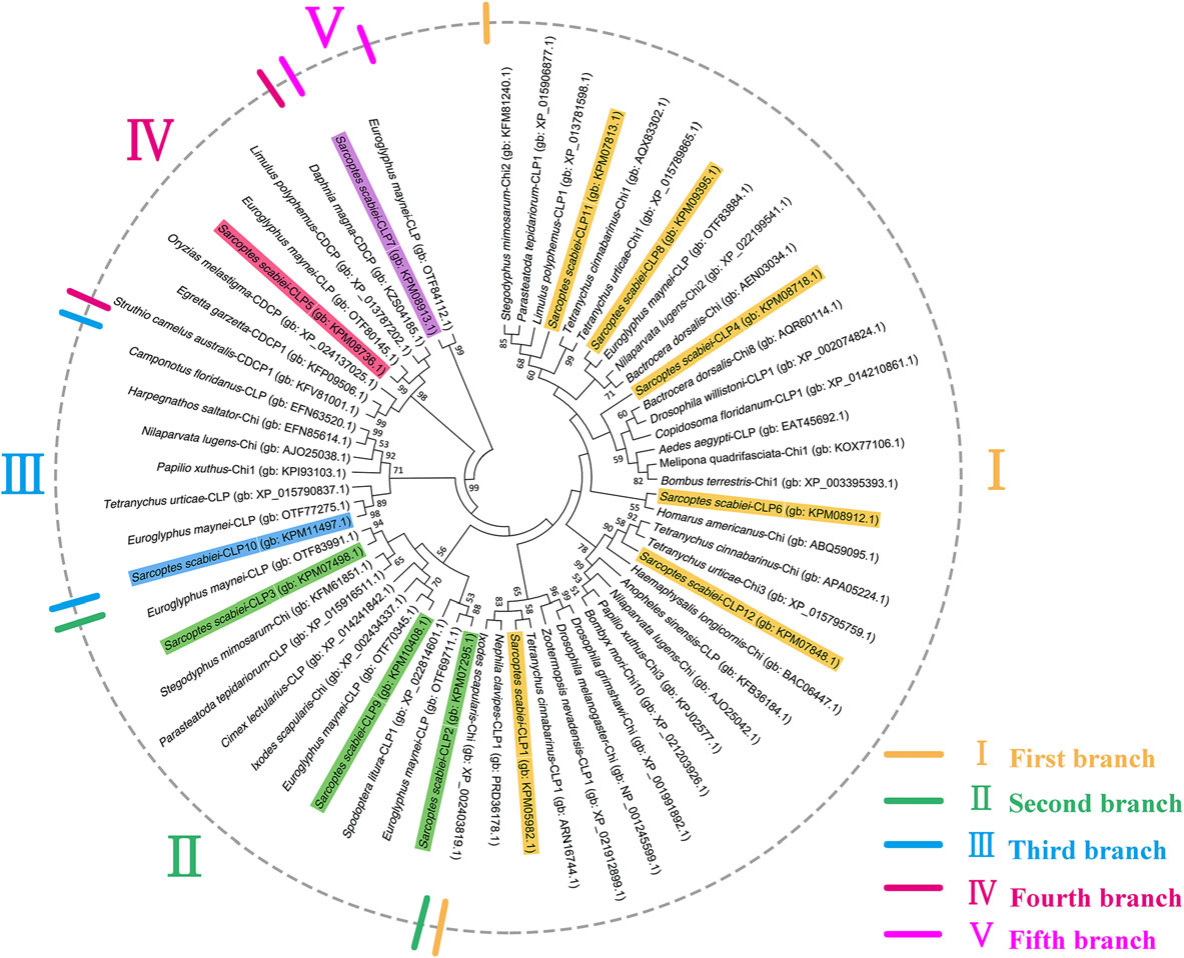
Phylogenetic relationships between SsCLPs and CLPs and chitinases from other species. The unrooted phylogenetic tree was constructed using CLP and chitinase sequences from approximately 40 species by the maximum likelihood (ML) method in MEGA software. CLP sequences or chitinase sequences were used in the tree (with their GenBank or SwissProt accession numbers). The number behind the protein acronym (for example Chi1, CLP2, CLP3, etc.) is from the NCBI database which represents more than one kind of protein in the species. Accession numbers are as follows: *Stegodyphus mimosarum*-Chi2 (gb: KFM81240.1); *Parasteatoda tepidariorum*-CLP1 (gb: XP_015906877.1); *Limulus polyphemus*-CLP1 (gb: XP_013781598.1); *S. scabiei*-CLP11 (gb: KPM07813.1); *Tetranychus cinnabarinus*-Chi1 (gb: AQX83302.1); *Tetranychus urticae*-Chi1 (gb: XP_015789865.1); *S. scabiei*-CLP8 (gb: KPM09395.1); *E. maynei*-CLP (gb: OTF83884.1); *Nilaparvata lugens*-Chi2 (gb: XP_022199541.1); *Bactrocera dorsalis*-Chi (gb: AEN03034.1); *S. scabiei*-CLP4 (gb: KPM08718.1); *B. dorsalis*-Chi8 (gb: AQR60114.1); *Drosophila willistoni*-CLP1 (gb: XP_002074824.1); *Copidosoma floridanum*-CLP1 (gb: XP_014210861.1); *Aedes aegypti*-CLP (gb: EAT45692.1); *Melipona quadrifasciata*-Chi1 (gb: KOX77106.1); *Bombus terrestris*-Chi1 (gb: XP_003395393.1); *S. scabiei*-CLP6 (gb: KPM08912.1); *Homarus americanus*-Chi (gb: ABQ59095.1); *T. cinnabarinus*-Chi (gb: APA05224.1); *T. urticae*-Chi3 (gb: XP_015795759.1); *S. scabiei*-CLP12 (gb: KPM07848.1); *Haemaphysalis longicornis*-Chi (gb: BAC06447.1); *Anopheles sinensis*-CLP (gb: KFB36184.1); *N. lugens*-Chi (gb: AJO25042.1); *Papilio xuthus*-Chi3 (gb: KPJ02577.1); *Bombyx mori*-Chi10 (gb: XP_021203926.1); *Drosophila grimshawi*-Chi (gb: XP_001991892.1); *Drosophila melanogaster*-Chi (gb: NP_001245599.1); *Zootermopsis nevadensis*-CLP1 (gb: XP_021912899.1); *T. cinnabarinus*-CLP1 (gb: ARN16744.1); *S. scabiei*-CLP1 (gb: KPM05982.1); *Nephila clavipes*-CLP1 (gb: PRD36178.1); *Ixodes scapularis*-Chi (gb: XP_002403819.1); *S. scabiei*-CLP2 (gb: KPM07295.1); *E. maynei*-CLP (gb: OTF69711.1) ; *Spodoptera litura*-CLP1 (gb: XP_022814601.1); *S. scabiei*-CLP9 (gb: KPM10408.1); *E. maynei*-CLP (gb:OTF70345.1); *I. scapularis*-Chi (gb: XP_002434337.1); *Cimex lectularius*-CLP (gb: XP_014241842.1); *P. tepidariorum*-CLP (gb: XP_015916511.1); *S. mimosarum*-Chi (gb: KFM61851.1); *S. scabiei*-CLP3 (gb: KPM07498.1 ); *E. maynei*-CLP (gb: OTF83991.1); *S. scabiei*-CLP10 (gb: KPM11497.1); *E. maynei*-CLP (gb: OTF77275.1); *T. urticae*-CLP (gb: XP_015790837.1); *P. xuthus*-Chi1 (gb: KPI93103.1); *N. lugens*-Chi (gb: AJO25038.1); *Harpegnathos saltator*-Chi (gb: EFN85614.1); *Camponotus floridanus*-CLP (gb: EFN63520.1); *Struthio camelus australis*-CDCP1 (gb: KFV81001.1 ) CDCP1; *Egretta garzetta*-CDCP1 (gb: KFP09506.1); *Oryzias melastigma*-CDCP (gb: XP_024137025.1); *S. scabiei*-CLP5 (gb: KPM08736.1); *E. maynei*-CLP (gb: OTF80145.1); *L. polyphemus*-CDCP (gb: XP_013787202.1); *Daphnia magna*-CDCP (gb: KZS04185.1); *S. scabiei*-CLP7 (gb: KPM08913.1) and *E. maynei*-CLP (gb: OTF84112.1). *Abbreviations*: gb, GenBank or SwissProt accession numbers; Chi, chitinase; CLP, chitinase-like protein; CDCP, chitinase domain-containing protein

The SsCLP5 DNA complete sequence length is 1701 bp containing an open reading frame (ORF) of 1251 bp and encoding a putative protein with 416 amino acid residues. Based on the results of multiple species alignment and epitope prediction analysis, the highly similar region (nucleotides 457 to 1251 of the ORF) of SsCLP5 was amplified. The amplified region of SsCLP5 includes a 795 bp ORF encoding a putative protein of 264 amino acid residues (~30.9 kDa) with a pI of 8.92 that lacks signal peptide or transmembrane domains, and has strong hydrophilicity. The SsCLP5 protein sequence of *S. scabiei*, together with its 11 homologues, were subjected to phylogenetic analysis. The topological tree divided these 12 SsCLPs into five different clades. Specifically, SsCLP1, SsCLP4, SsCLP6, SsCLP8, SsCLP11 and SsCLP12 showed a close evolutionary relationship and formed the first branch, and SsCLP2, SsCLP3 and SsCLP9 formed the second branch (Fig. 1). However, others were presented separately in three branches. The SsCLP5 characterized here was classified into the fourth branch and shared 99% bootstrap values with the CLP from *Euroglyphus maynei* (Fig. 1).

### Production and characterization of recombinant SsCLP5

**Fig. 2:**
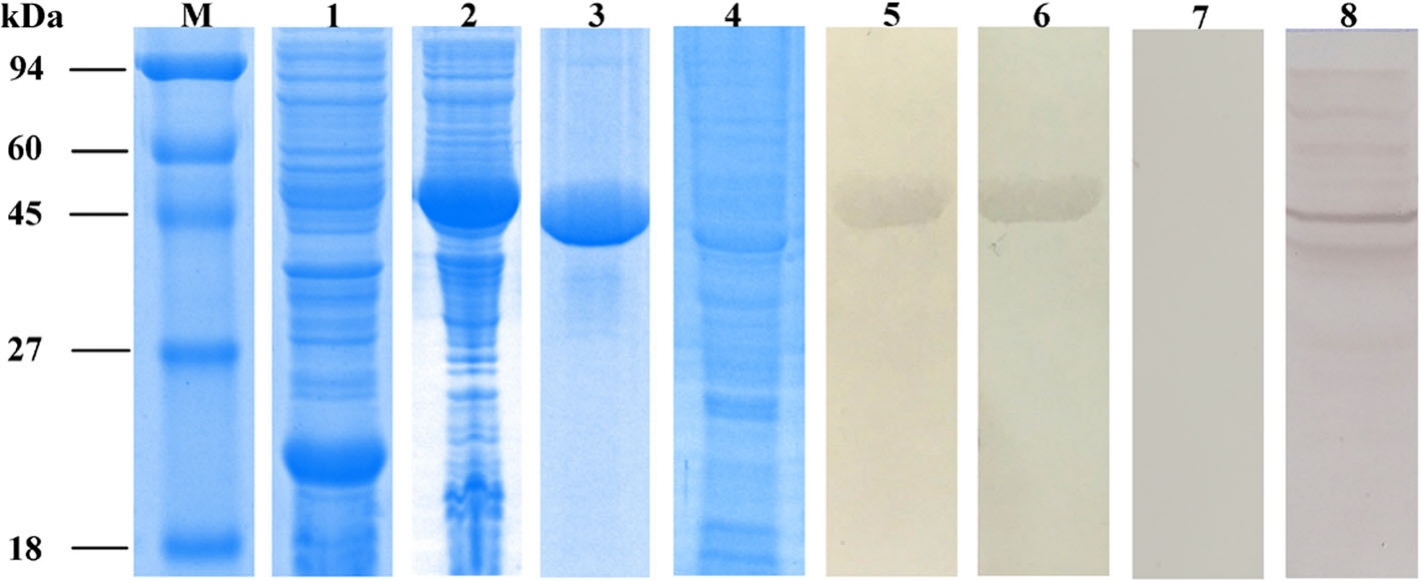
SDS-PAGE and western blotting of SsCLP5. Lane M: protein molecular weight markers (in kDa); Lane 1: pet-32a (+) expression vector protein; Lane 2: non-purified rSsCLP5 [soluble protein expressed from *Escherichia* coli BL21 (DE3)]; Lane 3: purified rSsCLP5; Lane 4: total crude proteins from *S. scabiei*; Lane 5: purified rSsCLP5 detected by serum (diluted 1:120 with 0.01 M PBS) from a rabbit naturally infested with *S. scabiei* (experimental group); Lane 6: purified rSsCLP5 detected by serum (diluted 1:120 with 0.01 M PBS) from a rSsCLP5-vaccinated rabbit (positive control); Lane 7: purified rSsCLP5 detected by pre-immune rabbit serum (diluted 1:120 with 0.01 M PBS; negative control); Lane 8: total crude proteins detected with rabbit anti-rSsCLP5 serum (diluted 1:120 with 0.01 M PBS). Samples derived from the same experiment and gels/blots were processed in parallel

The 795 bp ORF sequence was successfully cloned and then sub-cloned into the pet-32a (+) expression vector (Invitrogen) and expressed in *Escherichia coli* BL21 (DE3) cells. Protein expression level of SsCLP5 peaked at 6 h following induction with 1 mM IPTG. Recombinant SsCLP5 was expressed as soluble protein with a molecular mass of approximately 49 kDa (including vector-encoded amino acids; Fig. 2, Lane 2). The purification of the soluble protein was accomplished by immobilized metal affinity chromatography under denaturing conditions according to the manufacturer’s instructions. After purification and concentration, the protein was assessed by 12% sodium dodecyl sulfate polyacrylamide gel electrophoresis (SDS-PAGE). Purified rSsCLP5 migrated as a single band at the predicted size of approximately 49 kDa (Fig. 2, Lane 3).

The immunoreactivity of rSsCLP5 was examined by immunoblot. Serum samples from rabbits naturally infested with *S. scabiei* (experimental group) and serum from rabbits vaccinated with rSsCLP5 both bound the ~49 kDa protein in the antigen preparation, signifying strong reactivity and good antigenicity of the SsCLP5 recombinant protein (Fig. 2, Lanes 5, 6). The serum from pre-immune rabbit (negative control) did not bind any protein component in the antigen preparation (Fig. 2, Lane 7). For the internal reference, ~45 kDa (416 amino acid residues) protein from the total mite extract was bound by the serum from the rabbits vaccinated with rSsCLP5 in experimental group (Fig. 2, Lane 8).

### Localization of SsCLP5 in mites

**Fig. 3:**
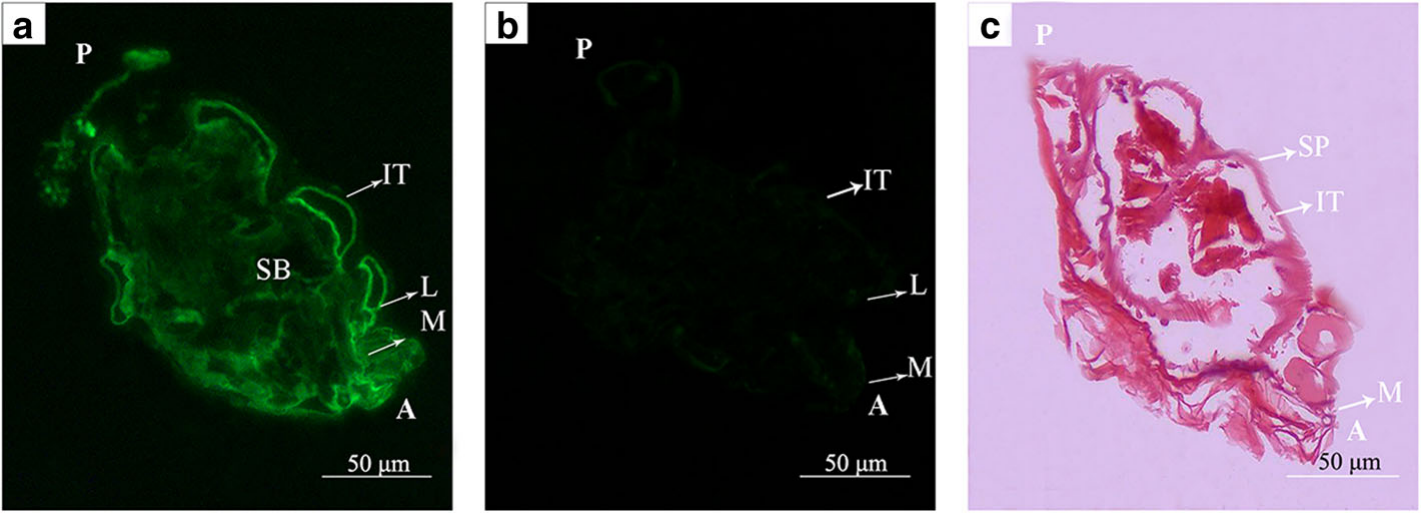
Immunolocalization of SsCLP5 in *S. scabiei* tissue (**a**, **b**) and H & E sections of *S. scabiei*. **a** *S. scabiei* incubated with anti-rSsCLP5 antibody as the primary antibody. **b** Negative control (antibody of the pre-immune rabbit serum), **c** H & E sections of *S. scabiei*. *Abbreviations*: A, anterior end of mite; P, posterior end of mite; IT, integument of the exoskeleton; M, mouthparts; L, legs; SP, spicules; SB, stomach blocks

Fluorescence immunohistochemistry was performed to examine SsCLP5 localization in mites. There was a wide distribution in the chewing mouthpart, legs and exoskeleton of *S. scabiei*, especially the outer layer of the exoskeleton (Fig. 3a). There was no fluorescence signal in the control group, which was treated with primary antibody from pre-immune rabbit serum (Fig. 3b). In addition, the hematoxylin-eosin (H & E) stained tissue sections of scabies mite including mouthparts, spicules and the integument of exoskeleton are depicted in Fig. 3c.

### Vaccination trial, lesion areas and mite burden

**Fig. 4:**
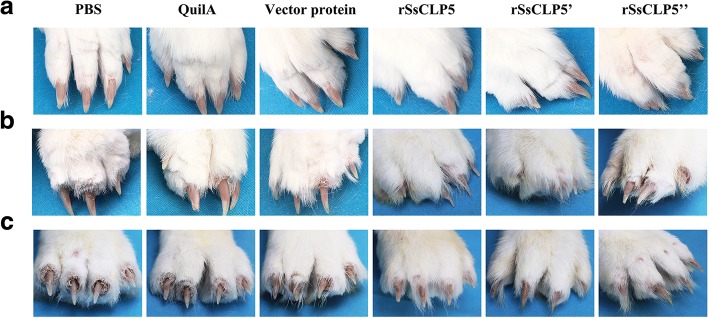
Clinical symptoms in rabbits before, two and four weeks post-challenge. Series **a** shows normal hind limbs in rabbits before challenge, series **b** shows clinical symptoms of hind limbs in rabbits after challenge with scabies mites for two weeks, and series **c** shows clinical symptoms of hind limbs in rabbits after challenge with scabies mites for four weeks. *Key*: PBS, immunized with 1 ml 0.01 M PBS each time; Quil A, immunized with 1 ml QuilA saponin at concentration of 1 mg/ml (dissolved in PBS) each time; vector protein, immunized with 100 μg (first time) and 200 μg (second time) purified pET32a(+) vector protein with 1 ml QuilA saponin at concentration of 1 mg/ml (dissolved in PBS); rSsCLP5, rSsCLP5’, and rSsCLP5” refer to the test groups immunized with 100 μg then 200 μg purified rSsCLP5 protein with 1 ml QuilA saponin at concentration of 1 mg/ml (dissolved in PBS) at the first and second immunizations

**Fig. 5:**
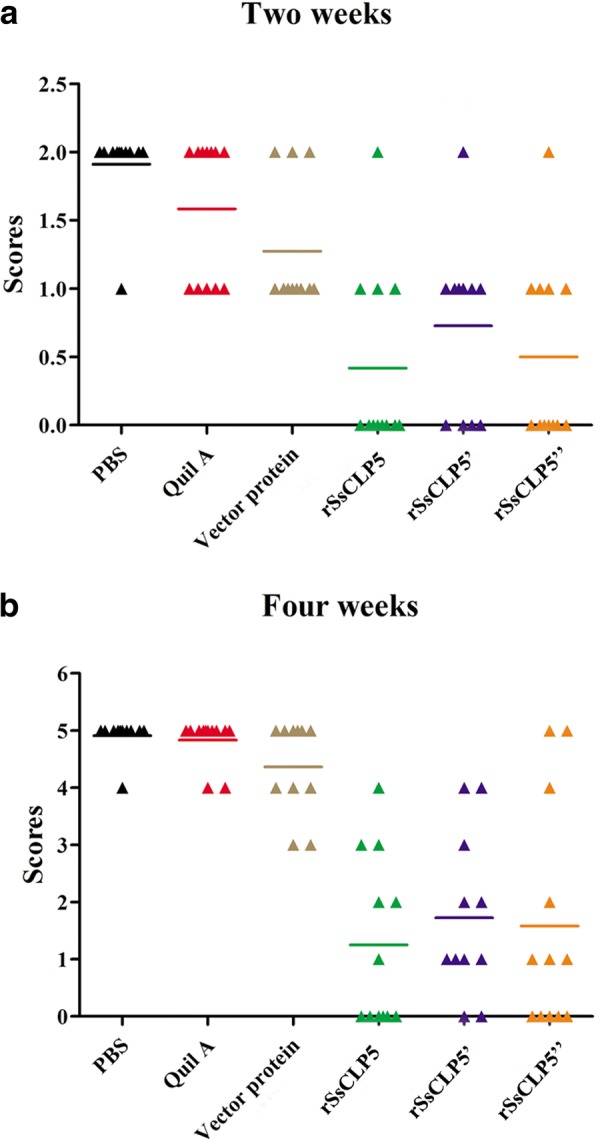
Inflammatory reaction grades and skin lesions at two (**a**) and four weeks (**b**) post-challenge. The colored lines indicate the group average. *Key*: PBS, immunized with 1 ml 0.01 M PBS each time; Quil A, immunized with 1 ml QuilA saponin at concentration of 1 mg/ml (dissolved in PBS) each time; vector protein, immunized with 100 μg (first time) and 200 μg (second time) purified pET32a(+) vector protein with 1 ml QuilA saponin at concentration of 1 mg/ml (dissolved in PBS); rSsCLP5, rSsCLP5’, and rSsCLP5” refer to the test groups immunized with 100 μg then 200 μg purified rSsCLP5 protein with 1 ml QuilA saponin at concentration of 1 mg/ml (dissolved in PBS) at the first and second immunizations. The grades of inflammatory reaction and skin lesions in the rSsCLP5 immunized groups (rSsCLP5, rSsCLP5’, and rSsCLP5”) were significantly lower compared to the control groups at week 2 post-challenge (*F*_(5,63)_ = 13.575, *P* < 0.0001) and at week 4 post-challenge (*F*_(5,63)_ = 23.38, *P* < 0.0001)

The protective effects of the rSsCLP5-based vaccine were assessed by grading the inflammatory response and measurement of the lesion area after challenge. Five days post-challenge, inflammation in most of the infested hind feet and itching symptoms were observed in rabbits in all six groups. Ten days post-challenge, the clinical symptoms in 74.3% (26/35; one death) of the rabbits in the three test groups had resolved and showed no significant differences compared to the challenge-free rabbits. However, the three control groups showed more severe clinical symptoms than that of the test groups at 2 weeks post-challenge, including significant inflammation and itching. Meanwhile, some rabbits in the control groups began to produce crusts (Fig. 4b). With the progression of challenge infestation till 4 weeks, the differences in clinical symptoms between the control and test groups were more significant (Fig. 4c). As shown by the grades of the inflammatory reaction in Fig. 5a, each of the control groups had mean scores > 1 at 2 weeks post-challenge, and even the mean score of the PBS group was close to 2. Conversely, the test groups all had mean scores < 1. After 4 weeks post-challenge, the rabbits in the control groups presented severe scabies with mean scores > 4; however, only 9 rabbits had higher scores (≥ 3 points) in the test groups (Fig. 5b). The scores of the inflammatory reaction in the control groups (two deaths) were significantly higher compared to the test groups (one death) at 4 weeks post-challenge (*F*_(5,63)_ = 23.38, *P* < 0.0001), and the differences became more apparent over the course of the infestation.

**Fig. 6:**
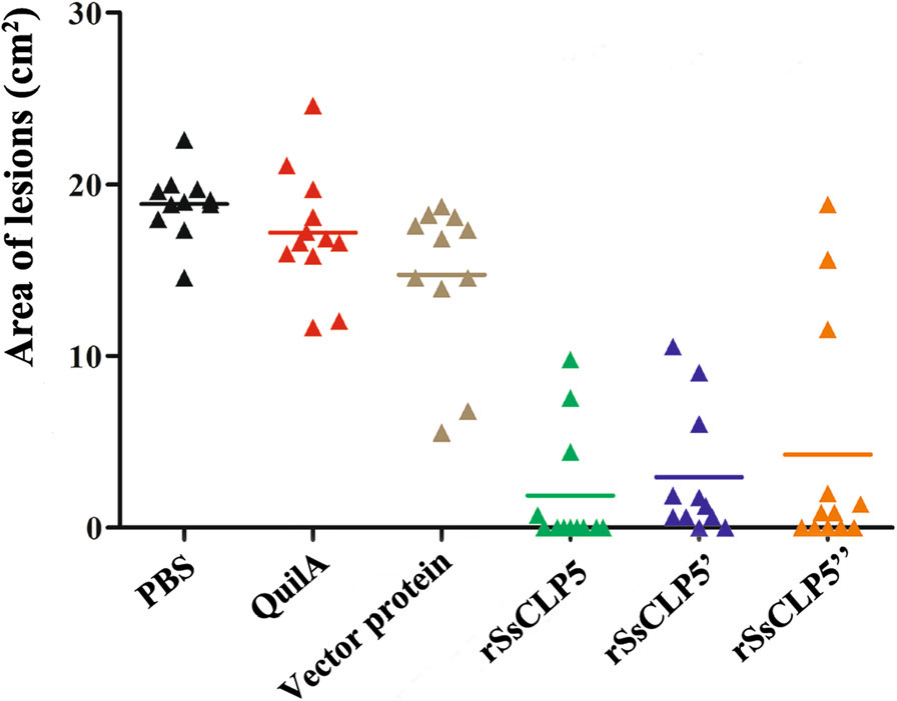
Area of lesions on rabbit hind limbs at four weeks post-challenge. The colored lines indicate the group average. *Key*: PBS, immunized with 1 ml 0.01 M PBS each time; Quil A, immunized with 1 ml QuilA saponin at concentration of 1 mg/ml (dissolved in PBS) each time; vector protein, immunized with 100 μg (first time) and 200 μg (second time) purified pET32a(+) vector protein with 1 ml QuilA saponin at concentration of 1 mg/ml (dissolved in PBS); rSsCLP5, rSsCLP5’, and rSsCLP5” refer to the test groups immunized with 100 μg then 200 μg purified rSsCLP5 protein with 1 ml QuilA saponin at concentration of 1 mg/ml (dissolved in PBS) at the first and second immunizations. The values of area of lesions in the rSsCLP5 immunized groups (rSsCLP5, rSsCLP5’, and rSsCLP5”) were significantly lower compared to PBS control, Quil A control and vector protein control at week 4 post-challenge (*F*_(5,63)_ = 36.99, *P* < 0.0001)

**Fig. 7:**
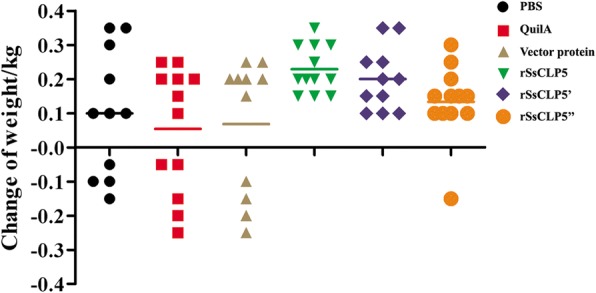
Weight changes of the rabbits at week 4 post-challenge compared to prior immunization. The colored lines indicate the group average. *Key*: PBS, immunized with 1 ml 0.01 M PBS each time; Quil A, immunized with 1 ml QuilA saponin at concentration of 1 mg/ml (dissolved in PBS) each time; vector protein, immunized with 100 μg (first time) and 200 μg (second time) purified pET32a(+) vector protein with 1 ml QuilA saponin at concentration of 1 mg/ml (dissolved in PBS); rSsCLP5, rSsCLP5’, and rSsCLP5” refer to the test groups immunized with 100 μg then 200 μg purified rSsCLP protein with 1 ml QuilA saponin at concentration of 1 mg/ml (dissolved in PBS) at the first and second immunizations. The weight changes of rabbits in the rSsCLP5 immunized groups (rSsCLP5 and rSsCLP5’) were significantly higher compared to PBS control, Quil A control and vector protein control at week 4 post-challenge (*F*_(5,63)_ = 2.689, *P* = 0.029)

**Fig. 8:**
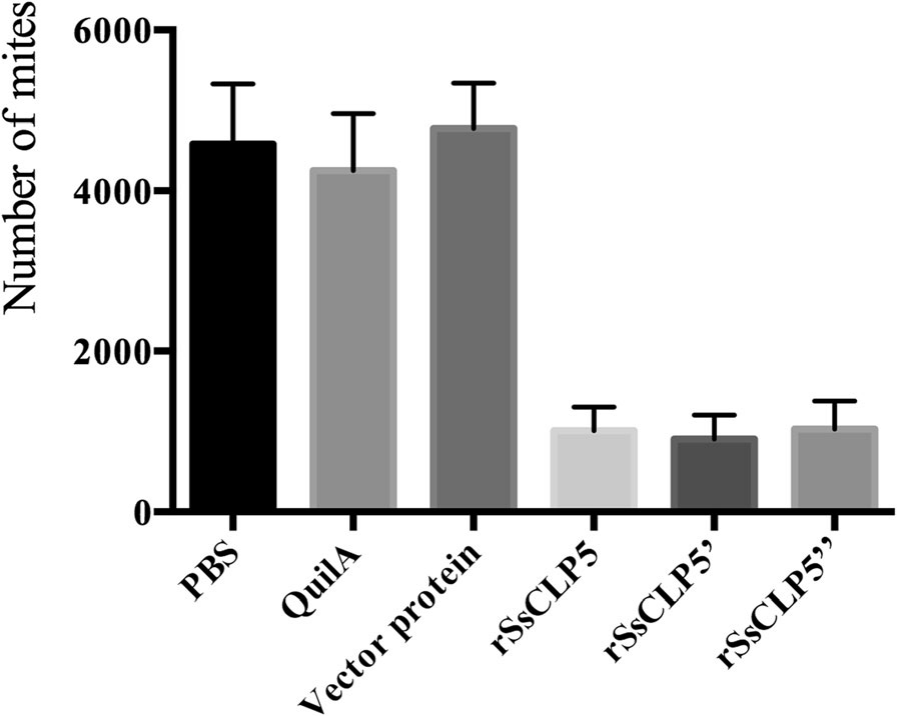
Mite burden in rabbits at week 4 post-challenge. *Key*: PBS, immunized with 1 ml 0.01 M PBS each time; Quil A, immunized with 1 ml QuilA saponin at concentration of 1 mg/ml (dissolved in PBS) each time; vector protein, immunized with 100 μg (first time) and 200 μg (second time) purified pET32a(+) vector protein with 1 ml QuilA saponin at concentration of 1 mg/ml (dissolved in PBS); rSsCLP5, rSsCLP5’, and rSsCLP5” refer to the test groups immunized with 100 μg then 200 μg purified rSsCLP protein with 1 ml QuilA saponin at concentration of 1 mg/ml (dissolved in PBS) at the first and second immunizations. Data columns correspond to the mean values; error bars represent the standard error. The mean values of mite burden in test groups immunized with rSsCLP5 were significantly lower compared to the control groups at week 4 post-challenge (*F*_(5,63)_ = 39.354, *P* < 0.0001)

As for the lesion area at week 4 post-challenge, our results showed that the mean values of lesion areas in PBS, QuilA, and vector protein groups were 18.86 cm^2^, 17.19 cm^2^, and 14.74 cm^2^, respectively. However, 74.3% of rabbits (26/35) immunized with rSsCLP5 had no detectable lesions or very sparse horny hyperplasia (Fig. 6). Moreover, the levels of skin lesions in control groups were significantly higher compared to the rSsCLP5 immunized groups (*F*_(5,63)_ = 36.99, *P* < 0.0001). At the end of the trial, nearly 80% of the 69 rabbits showed different degrees of body weight gain from 0.1 to 0.35 kg at 4 weeks post-challenge compared to prior immunization weights (Fig. 7). Some of rabbits (13 rabbits, nearly 20%) from three control groups showed weight loss ranging from 0.05 to 0.25 kg; however, in the rSsCLP5 immunized group, weight loss (0.15 kg of body weight) was only observed in one rabbit (Fig. 7). Further observations revealed that the scabies did not spread to forelimbs at the end of the vaccination trial. We also analyzed the mite burden in the hind limbs, which serves as an indicator of the protective value of the rSsCLP5-based vaccine (Fig. 8). The mean number of mites was significantly higher (*F*_(5,63)_ = 39.354, *P* < 0.0001) in the control groups (more than 4000 mites/rabbit) compared to rSsCLP5 vaccinated groups (nearly 1000 mites/rabbit).

### Antibody responses

**Fig. 9:**
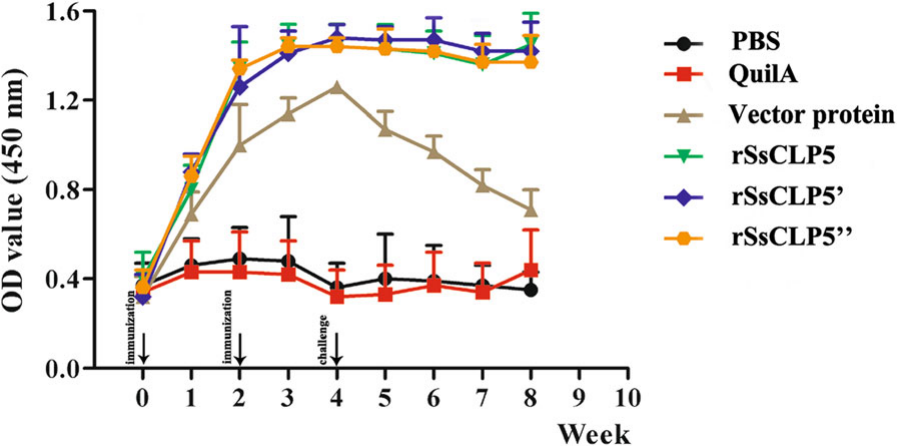
Variation of specific IgG antibody levels in sera of immunized rabbits as detected by ELISA. Rabbits were immunized twice. *Key*: PBS, immunized with 1 ml 0.01 M PBS each time; Quil A, immunized with 1 ml QuilA saponin at concentration of 1 mg/ml (dissolved in PBS) each time; vector protein, immunized with 100 μg (first time) and 200 μg (second time) purified pET32a(+) vector protein with 1 ml QuilA saponin at concentration of 1 mg/ml (dissolved in PBS); rSsCLP5, rSsCLP5’, and rSsCLP5” refer to the test groups immunized with 100 μg then 200 μg purified rSsCLP5 protein with 1 ml QuilA saponin at concentration of 1 mg/ml (dissolved in PBS) at the first and second immunizations. Data points correspond to the mean values; error bars represent the standard error. The groups immunized with the rSsCLP5 had significantly higher IgG levels compared to the control groups (*F*_(5,63)_ = 37.285, *P* < 0.0001) from week 1 after first immunization to the end of the experiment

The optimal conditions for the rSsCLP5-based indirect ELISA were determined to be 4 μg/ml of rSsCLP5 protein, a 1:120 serum dilution, and a 1:3000 dilution of secondary antibody. Specific IgG antibody was detected by rSsCLP5-based indirect ELISA as previously described [37]. The results showed that the specific IgG antibodies increased at one week after the first vaccination in the rSsCLP5 vaccine groups, and levels were significantly higher than the antibody levels of three control groups (*F*_(5,63)_ = 37.285, *P* < 0.0001) (Fig. 9). At the same time, the IgG antibody levels observed in the vector protein group also increased, but at lower levels than in the rSsCLP5 vaccine groups. Two weeks after the second immunization, the specific IgG antibodies levels increased to the highest value (OD 450 nm ~1.4) and stabilized at high levels after the challenge in the three vaccinated groups. The IgG levels in the vaccinated groups were also significantly higher than in the controls at two weeks after the second immunization (*F*_(5,63)_ = 614.491, *P* < 0.0001). In our pilot experiments, the high antibody levels increased to OD 450 nm ~1.8 and were stable for more than three months (data not shown). In the QuilA saponin and PBS control groups, the OD 450 nm values of specific IgG remained low throughout the experiment (Fig. 9). Additionally, the low levels antibody of the vector protein group rapidly decreased during the experimental period (Fig. 9).

## Discussion

Chitinases and CLPs are a diverse group of proteins, as shown in the phylogenetic tree, with more than five categories of enzymes and proteins. It has been reported that these enzymes and proteins have a complex array of functions in mites and hosts, including roles in ecdysis, digestion, allergic reactions, immune response and resistance to infection, among others [42–44]. In western blotting experiments, the serum from rabbits infested with *S. scabiei* produced a strong signal on the blot, indicating that the SsCLP5 plays an important role in eliciting an immune response during natural infestation. When we used serum from rabbits inoculated with rSsCLP5 to probe the total body extracts of the mites, we detected the antigen signal at ~45 kDa, suggesting that the protein is high expression in mites as previously reported [26].

Fluorescence immunohistochemistry assays showed strong fluorescence signals of SsCLP5 present in the exoskeleton, chewing mouthpart and legs of *S. scabiei*. Signals were especially strong in the outer layer of the exoskeleton, suggesting that the SsCLP5 is highly expressed in the exoskeleton of scabies mites. Thus, it is possible that this protein presents in the epidermis of the rabbit after the death and lysis of mites, and the further recurrent immune responses in the host cannot be entirely ruled out. It has been reported that homologous proteins in other species are immune-related proteins that can cause different levels of immune responses in hosts [45–48]. The high expression of SsCLP5 supports its potential as a candidate vaccine protein against *S. scabiei*.

During the immunization trial, some rabbits in the mite control group examined at 4 weeks post-challenge showed severe scabies and weight loss, which is associated with scabies as a chronic wasting disease. Previous reports have similarly observed that severe scabies results in decreased body weight in rabbits and other animals, both in the wild and in laboratory animal models [49, 50]. In our study, the majority of rabbits vaccinated with rSsCLP5 and challenged with the scabies mite displayed weight gain due to the protective effects of rSsCLP5 and because the rabbits were in a growth and development stage. Interestingly, previous work has shown that re-infested animals show a reduced mite burden as compared to the initial mite infestation [20, 21, 51]. These reports demonstrate that mites or mite secretions induce immune protection in the host. Additionally, immunization of rabbits with whole body extracts of *S. scabiei* (var. *canis*) induced antibodies to more antigens than infestation with the mite [52]. Based on these and other recombinant protein studies [22, 24], we chose to immunize rabbits twice with an increasing dose of rSsCLP5 to obtain higher antibody levels. We created a grading scale to assess the severity of skin lesions caused by the mites. We observed that rabbits immunized with the rSsCLP5 acquired immune protection and the majority of the rabbits (74.3%) had no detectable lesions showing successful development of resistance against the scabies at four weeks post-challenge. We observed significant differences between the groups immunized with rSsCLP5 and control groups with respect to inflammation and lesion area at week 2 post-challenge (*F*_(5,63)_ = 13.575, *P* < 0.0001) and at week 4 post-challenge (*F*_(5,63)_ = 23.38, *P* < 0.0001). Approximately five days after the challenge, a comparison of all groups showed that the skin of the hind feet in all of the rabbits was severely inflamed. These symptoms gradually subsided by day 10 post-infestation. This could be associated with the release of substances that induce inflammatory and immune responses by the mites [53–55] and/or the inhibition of early immune responses by *S. scabiei* by downregulating the expression of pro-inflammatory mediators and cytokines [54, 56–58]. Studies of the life-cycle and infestation of *S. scabiei* found that most mites (*S. scabiei* var. *canis*) rapidly initiate penetration of the skin of the rabbit host [59] and develop from egg to adult in 10–13 days [60]. Immunomodulation by the mites, which appears to impact the development of the immune response during infestation in hosts, might explain why some animals fail to develop resistance to re-infestation by *S. scabiei* [61, 62]. In our study, although we observed inflammation and lesions in some of the rabbits in the vaccinated group, the extent was lower than that observed in the infected control groups. On week 4 post-challenge, the mean values of the mite burden in test groups were significantly lower compared to the control groups (*F*_(5,63)_ = 39.354, *P* < 0.0001). These differences in mite burden between the control and treated groups may be attributed to the immune protection induced by rSsCLP5 in the latter groups. Furthermore, immunization of rabbits with rSsCLP5 might have affected the ability of mites to infect, grow and reproduce.

A previous study found no protective effect when vaccinating goats with the extract of scabies mites [51]. Vaccination trials of rabbits with the recombinant antigens Ssag1 and Ssag2 (*S. scabiei* var. *hominis*) produced antibodies, but the rabbits displayed no protection or reduced mite burden [63]. In this study, we observed that the IgG levels in the adjuvant group and the PBS control group did not change significantly and maintained relatively stable levels after immunization. In the group immunized with the protein from the empty vector, antibody levels peaked 2 weeks after the second immunization. However, the antibody levels showed a very rapid decrease after a short time with no protective effects, indicating that the vector protein had little or no effect on the immunization effects of rSsCLP5 and production of the specific antibody. Additionally, both the inflammatory grades and lesion areas of animals in the vector protein group were higher than in the rSsCLP5 vaccinated groups. In the three test groups, the antibody levels reached the highest values (OD450 ~1.4) 2 weeks after the second immunization with rSsCLP5, with levels persisting for a longer period of time. Previous studies found that vaccinated rabbits exhibited high levels of IgG and increased total IgE levels when immunized with a mix of the recombinant antigens Ssλ20ΔB3 and GST-Ssλ15 (*S. scabiei* var. hominis); however, the rabbits had no protection against mite challenge [64]. In contrast, our study found high levels of antibodies to be produced and, importantly, our results showed that rSsCLP5 could induce immune protection. Three vaccinated groups showed different levels of protection against scabies mite infestation. The majority (74.3%) of rabbits did not show clinical symptoms of scabies after challenge with *S. scabiei*; however, some did show different degrees of infestation. The degree of infestation was not as serious as in the unvaccinated control groups and furthermore, rabbits will not develop scabies with an extremely small number of mites.

Taken together, our findings support the further testing of a rSsCLP5-based vaccine for *S. scabiei*. Future work focusing on optimizing parameters such as the protein concentrations required for an effective vaccine, the use of adjuvants, the number of immunizations, and the time interval between different injections will be of great value. In particular, investigations providing insights regarding the term protection of these candidate vaccine proteins are awaited.

## Conclusions

In summary, SsCLP5 was identified and characterized in the present study. Furthermore, we evaluated rSsCLP5 as candidate vaccine protein for anti-mite protection in rabbits. Importantly, our data demonstrate that rSsCLP5 is a promising candidate for a recombinant protein-based vaccine against *S. scabiei*. This study also provides a method for studying scabies vaccine using rabbit as an animal model and a basis for screening more effective candidate proteins.

## Additional file

Additional file 1: **Table S1.** Number of scabies mite at week 4 post-challenge. (PDF 48 kb)

## Abbreviations

CLPs: Chitinase-like proteins
ELISA: Enzyme-linked immunosorbent assay
HRP: Horseradish peroxidase
IPTG: Isopropyl-beta-D-thiogalactopyranoside
PBS: Phosphate-buffered saline
rSsCLP5: Recombinant *S. scabiei* chitinase-like protein 5
SsCLP5: *S. scabiei* chitinase-like protein 5
TBST: Tris-buffered saline and Tween 20
