# Supplementary material for: A chitinase-like protein from Sarcoptes scabiei as a candidate anti-mite vaccine that contributes to immune protection in rabbits
Source: Parasit Vectors. 2018 Nov 20;11:599. doi: 10.1186/s13071-018-3184-y (PMC6245717; doi:10.1186/s13071-018-3184-y)
Supplement: Supplementary file 1 — Table S1. Number of scabies mite at week 4 post-challenge. (PDF 48 kb) [file 13071_2018_3184_MOESM1_ESM.pdf]

Additional file 1

Table S1 Number of scabies mite at week 4 post-challenge

Rabbit	Group					
	PBS	QuilA	Vector protein	rSsCLP5	rSsCLP5’	rSsCLP5”
1	4656	3958	5142	814	636	1554
2	5212	4200	3850	1256	638	1124
3	0	3840	5254	626	1238	826
4	5742	4270	4254	938	712	996
5	4594	5158	4752	1444	1204	1750
6	3478	4062	4640	780	598	1436
7	3940	3960	4234	930	1460	714
8	3824	5024	5534	984	704	738
9	5752	3024	0	1048	756	844
10	4510	5696	5006	1586	1052	630
11	3976	3962	5140	942	996	754
12	4430	4146	5422	706	0	916
**Average**	**4556**	**4275**	**4839**	**1005**	**909**	**1024**

The number ‘0’ indicates the rabbit that died during the experiment.
